# Advanced glycation end products and protein carbonyl levels in plasma reveal sex-specific differences in Parkinson's and Alzheimer's disease

**DOI:** 10.1016/j.redox.2020.101546

**Published:** 2020-05-18

**Authors:** Amit Sharma, Daniela Weber, Jana Raupbach, Tikam Chand Dakal, Klaus Fließbach, Alfredo Ramirez, Tilman Grune, Ullrich Wüllner

**Affiliations:** a Department of Neurology, University Clinic Bonn, Bonn, Germany; b Department of Ophthalmology, University Clinic Bonn, Bonn, Germany; c Department of Molecular Toxicology, German Institute of Human Nutrition Potsdam, Rehbruecke (DIfE), 14558, Nuthetal, Germany; d Department of Biotechnology, Mohanlal Sukhadia University, Rajasthan, India; e Department of Neurodegenerative Diseases and Geriatric Psychiatry, University Hospital Bonn, Bonn, Germany; f German Center for Neurodegenerative Diseases (DZNE), Bonn, Germany; g Division of Neurogenetics and Molecular Psychiatry, Department of Psychiatry and Psychotherapy, Medical Faculty, University of Cologne, Cologne, Germany

**Keywords:** Advanced glycation end products, Protein carbonyls, Parkinson's diseases, Alzheimer's diseases, Oxidative stress, Neurodegeneration, Carboxyethyllysine, Carboxymethyllysine, Biomarker

## Abstract

Neurodegenerative diseases (NDD) such as Alzheimer's (AD) and Parkinson's disease (PD) are distinct clinical entities, however, the aggregation of key neuronal proteins, presumably leading to neuronal demise appears to represent a common mechanism. It has become evident, that advanced glycation end products (AGEs) trigger the accumulation of such modified proteins, which eventually contributes to pathological aspect of NDDs. Increased levels of AGEs are found in amyloid plaques in AD brains and in both advanced and early PD (incidental Lewy body disease). The molecular mechanisms by which AGE dependent modifications may modulate the susceptibility towards NDDs, however, remain enigmatic and it is unclear, whether AGEs may serve as biomarker of NDD. In the present study, we examined AGEs (CML: Carboxymethyllysine and CEL: Carboxyethyllysine), markers of oxidative stress and micronutrients in the plasma of PD and AD patients and controls. As compared to healthy controls, AD females displayed lower levels of CEL while higher levels of CML were found in AD and PD patients. A somewhat similar pattern was observed for protein carbonyls (PC), revealing lower values exclusively in AD females, whereas AD males displayed significantly higher values compared to healthy controls and PD. Sex-specific differences were also observed for other relevant markers such as malondialdehyde, 3-nitrotyrosine, γ -tocopherols, retinol, plasma proteins and α-carotene, while α-tocopherols, β-carotene, lutein/zeaxanthin, β-cryptoxanthin and lycopene showed no relevant association. Taken together, our study suggests yet unappreciated differences of the distribution of AGEs among the sexes in NDD. We therefore suggest to make a clear distinction between sexes when analyzing oxidative (AGEs)-related stress and carbonyl-related stress and vitamins.

## Highlights

• As compared to healthy controls, AD females displayed lower levels of CEL (carboxyethyllysine) while higher levels of CML (carboxymethyllysine) were found in AD and PD patients.
• A somewhat similar pattern was observed for protein carbonyls (PC), revealing lower values exclusively in AD females, whereas AD males displayed significantly higher values compared to healthy controls and PD. Presumed carbonlyated residues were identified the in the major proteins associated with neurodegenerative diseases.
• Sex-specific differences were also observed for other relevant markers such as malondialdehyde, 3-nitrotyrosine, γ-tocopherols, retinol, plasma proteins and α-carotene, while α-tocopherols, β-carotene, lutein/zeaxanthin, β-cryptoxanthin and lycopene showed no relevant association.

## Graphical abstract

**Figure:**
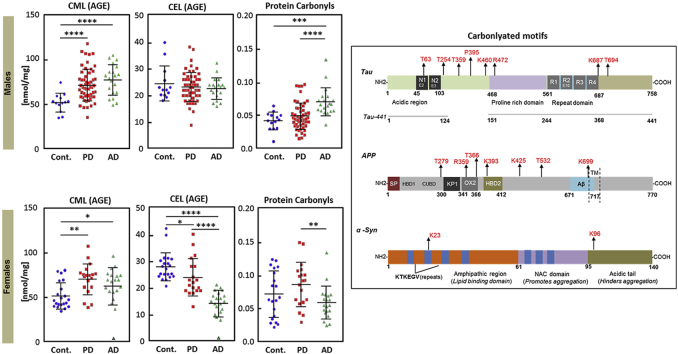

## 1 Introduction

More than three decades ago, oxidative stress and reactive oxygen species (ROS) have emerged as putative contributing factors in neurodegenerative diseases (NDD) [[1], [2], [3]]. Since then, considerable data have accumulated and several compounds with antioxidant properties (i.e. glutathione (GSH), vitamin C, vitamin E, coenzyme Q10, etc.) have been examined to reduce the impact of ROS-related neurodegenerative processes. By and large, the preclinical more than clinical studies indicate, that NDDs are characterized by (sometimes discrete) higher levels of oxidative stress and by lower levels of antioxidant defense markers in the brain and peripheral tissues [4].

Oxidative stress induces glycoxidation reactions, modifications of free amino groups in proteins, resulting in the generation of advanced lipoxidation and glycation end products (ALEs and AGEs). AGEs are major structural crosslinker that can transform soluble neurofilament proteins into insoluble aggregates. Glycation as a spontaneous age‐dependent posttranslational modification impacts the structure and function of several proteins. Glycation of α-synuclein with d-glucose for instance results in misfolding and aggregation and might modulate the initial formation of aggregates, and glycation has been detected at the periphery of Lewy bodies in Parkinson's disease (PD) patient's brains both at the early and advanced stages [5,6]. The presence of glycated forms of Aβ and tau in Alzheimer's disease (AD) and the immunohistochemical localization of AGEs suggests, that protein glycation acts as a common detrimental posttranslational modification of major NDD – associated proteins [7,8]. Recently, Li et al. synthesized the glycated Aβ in vitro and treated the hippocampal neurons and suggested that the glycated Aβ_42_ could be a new therapeutic target for AD [9].

As compared to other oxidative modifications, protein carbonyls (PCs) have unique stability and a wide range of downstream functional consequences. Primary (direct metal-catalyzed oxidation of amino acid side chains) and secondary (the addition of reactive aldehydes to amino acid side chains) protein carbonylation are recognized as marker of pathological reactive oxygen species (ROS) production in a variety of cell types and tissues [10,11]. Increased PC has consistently been reported in the hippocampus and neocortex in AD and in Lewy bodies of PD [[12], [13], [14]]. AGEs and PC as markers of oxidative stress may potentially also serve as biomarker when investigated and characterized in peripheral tissues. Plasma AGEs have been associated with cardiovascular events [15] and have also been studied in inflammatory diseases such as multiple sclerosis [16]. Several studies characterized AGEs and the related receptor (RAGE) in AD [17], but data in PD patients are scarce.

Herein, we determined baseline plasma levels of two well characterized AGEs (CEL: Carboxyethyllysine and CML: Carboxymethyllysine), protein carbonyls (PCs), other oxidative stress markers and fat-soluble micronutrients in PD, AD patients and healthy controls (HC). Additionally, we analyzed the presumed carbonlyated residues in the major proteins associated with NDD. We detected differential associations between NDD, sex and oxidative stress markers.

## 2 Material and methods

We have analyzed clinically well-defined PD (n = 70; age = 69 ± 4 years), AD (n = 40; age = 73 ± 5 years) and age matched healthy controls (n = 34; age = 68 ± 8 years). Hoehn and Hoehn and Yahr (HY) and the MMSE respectively were used to determine the level of disease severity (PD H&Y: 3,0 ± 0,7, range 2–4, median 3; AD MMSE: 20 ± 5, range 13–24, median 21). AD patients with MMSE >12 and <25 were selected for the analysis. The clinical profile for PD patients revealed no female and only few males (n = 9) with diabetes status, while such information was not available for AD samples. Prior to the analyses total protein concentration in plasma was measured by Bradford method. Protein-bound AGEs CML and CEL were analyzed as previously described^27^. In brief, an aliquot of 25 μl plasma was diluted with ultrapure water followed by reduction of early glycation products with sodium borohydride. Protein precipitation was performed with trichloroacetic acid. The protein pellet was spiked with 10 μl of internal standard containing 20 μM ^2^H_4_-CML and 20 μM ^2^H_4_-CEL. Protein hydrolysis was achieved by incubating the samples with 1 ml of 6 M HCl for 23 h at 110 °C. The hydrolyzed samples were evaporated to dryness with a vacuum concentrator (SpeedVac, Thermo Fisher Scientific, Germany) and the dry residue was dissolved in 100 μl eluent B (10 mM ammonium formiate). After centrifugation (4 °C, 10000 rpm, 10 min), an aliquot of 90 μl was subjected to UPLC-MS/MS analysis. UPLC analysis was performed with an Acquity Ultra Performance LC system coupled to a Waters Quattro Premier XE mass spectrometer (both Waters Corporation, Milford, MA, USA). Chromatographic conditions and detection parameters for AGE analysis are described in detail elsewhere [18]. All plasma samples were analyzed in duplicate.

The analysis of PC and 3-NT in plasma was performed by in-house ELISA by previously described method [19]. The following devices were used for both ELISA: Nunc Immuno 96 Microwell plate MaxiSorp (Sarstedt, Nuembrecht, Germany), Microplate Reader Infinite® M2000 PRO (Tecan, Crailsheim, Germany). Plasma MDA was determined after derivatization with thiobarbituric acid by RP-HPLC coupled with fluorescence detection according to Wong et al. with modifications described by Weber et al. [19,20]. Retinol, carotenoids (lutein, zeaxanthin, β-cryptoxanthin, lycopene, α-carotene and β-carotene) and tocopherols (α-tocopherol, γ-tocopherol) were measured in plasma by reversed-phase HPLC with UV and ﬂuorescence detection as previously described [21]. All reagents were of analytical or HPLC grade and purchased from Carl Roth (Karlsruhe, Germany) and Sigma-Aldrich (Steinheim, Germany). All HPLC devices were from Shimadzu and consists pump, autosampler, column heater and system controller (all from the LC-20A series), UV detector (SPD 20AV), a ﬂuorescence detector (RF 10A XL) and the chromatography workstation (LC Lab Solution).

As standard protocol, the values for AGEs, 3NT and PC were relative to the total plasma protein, while for other markers such as MDA and vitamins in the plasma measurements were performed after protein precipitation.

Statistical analyses were performed with SPSS Software (SPSS Inc., Chicago, IL, USA; Version 20.0.0) and differences between groups were considered to be significant at a P value of <0.05. The figures were generated with GraphPad Prism 5.0 (GraphPad Software, Inc., San Diego, CA) and the significance between groups was determined by nonparametric Mann-Whitney test.

In order to ascertain protein carbonylation which primarily occurs at as lysine, arginine, tyrosine and proline, we submitted the protein sequences (UniProt ID: P37840, P05067, P10636) to CarSPred tool [22], which is based on the datasets obtained from experimentally verified carbonylation sites from literature. Additionally, algorithms based on the evaluation of the minimum redundancy maximum relevance (mRMR), incremental feature selection (IFS) and weighted support vector machine (WSVM) feature were considered. The final result list was obtained with the default parameters and threshold value of 0.5.

## 3 Results and discussion

### 3.1 Patterns of oxidative stress biomarkers in AD and PD

**Fig. 1:**
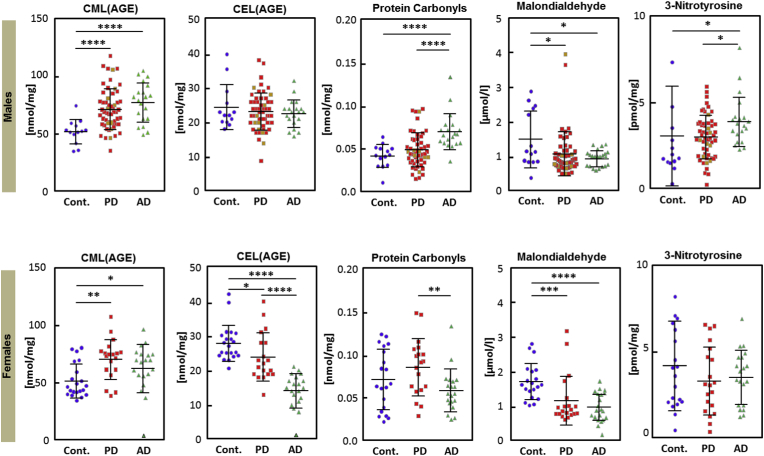
**Oxidative stress markers.** Plot showing plasma levels of advanced glycation end products [CML (carboxymethyllysine), CEL(carboxyethyllysine)], protein carbonyl, malondialdehyde and 3-nitrotyrosine. PD male samples with diabetes are marked in brown. Asterisks indicate level of statistical significance (*p < 0.05, **p < 0.01, ***p < 0.001, ****p < 0.0001). [Contl: controls; AD: Alzheimer's disease, PD: Parkinson's disease]. (For interpretation of the references to colour in this figure legend, the reader is referred to the web version of this article.)

Plasma levels of CML were found to be significantly higher in both PD and AD patients compared to age matched healthy controls (HC) (Males: Cont vs PD: p < 0,0001; Cont vs AD p < 0,0001; Females: Cont vs PD: p = 0,0032; Cont vs AD p = 0,0180, Mann–Whitney test) (Fig. 1, Supplementary Table 1). In contrast, AD females showed significantly lower CEL levels in comparison to healthy female controls and PD females (Females: Cont vs AD <0,0001, PD vs AD: <0,0001, Cont vs PD: <0,0113, Mann–Whitney test). AD females also show significantly lower CEL level compared to AD males (p < 0.0001, Mann–Whitney test). In PD, the oligomeric form of α-synuclein was found to be modified with CML in the substantia nigra in MPTP-induced C57BL/6 mouse model of parkinsonism [23]. Bar and colleagues also reported elevated levels of CML in CSF of AD patients as compared to the healthy controls [24]. Sex specific differences for CEL have been reported previously in multiple sclerosis, where authors observed significantly lower CEL plasma levels in HC females as compared to HC males and tended to be lower in male patients compared to the females [16]. The anti-oxidative effect of estrogens on CEL levels likely underlies this phenomenon. Also, in contrast to CEL (methylglyoxal pathway), multiple routes of CML formation (via Amadori product, direct reaction of glyoxal) have been-described in the literature. These data also imply that aging, specifically menopause, may play a peculiar role in observed sex -specific differences.

We found higher PC levels in the plasma of AD males compared to PD males and healthy controls (Males: Cont vs AD: p < 0,0001; PD vs AD p < 0,0001, Mann*–*Whitney test). AD females on the other hand showed significantly lower level than PD females (p = 0,0088, Mann*–*Whitney test), the difference to healthy controls did not reach statistical significance (Fig. 1). Previously, substantia nigra tissue from the postmortem brains of Parkinson's patients showed increased PC levels compared to the control brains or other brain regions [25]. Likewise, PC carbonyl levels were found to be significantly elevated compared to age-matched control in the postmortem cortex areas of sporadic, familiar Alzheimer's patients and those with mild cognitive impairment [13,26]. Thus, the observed changes in plasma most likely reflect changes in patient's brain. In addition, we examined whether the major AD and PD associated proteins are prone to carbonylation. We used a computational approach and identified putative carbonylation sites in Amyloid- β (K393, K425, K699, R359, T279, T366, T532), Tau (K460, K687, P359, R472, T63, T254, T395, T694) and α-synuclein (K23, K96) (Supplementary Fig. 1). However, it remains to be determined whether altered carbonylation levels are present at any of these residues or may have consequences for the protein-bound carbonylation/AGE products in the plasma. Taking into account that several APP (the β-amyloid precursor protein) mutations have been reported being associated with familial forms of AD and are localized within the flanking amyloid β-peptide (Aβ) and transmembrane (TM) domain of APP (aa647 to aa730, reviewed in Cacace et al. [27]). The carbonated site K699 predicted by us is located as overlapping residue of amyloid β-peptide (Aβ) (as extracellular domain) and TM domain, which is presumably a target for α-secretase and requires further attention. The impact of PD related medications such as l- DOPA on PCs remains controversial in the literature, with some studies suggesting that l-DOPA treatment may increase the PC [14], while others disagree [28,29]. In the present study, we did not account for l-DOPA usage. The observed changes are not sufficient to serve as a biomarker or indicator of disease in an individual patient, but in the groupwise comparison, the sex-specific differences underscore the necessity to divide groups of (NDD) patients into meaningful biological subgroups.

Among the redox biomarker under investigation, malondialdehyde (MDA), an indicator of lipid peroxidation, was reduced in both sexes of AD and PD as compared to HCs (Males: Cont vs PD: p < 0,0411; Cont vs AD p < 0,0394; Females: Cont vs PD: p = 0,0002; Cont vs AD p < 0,0001, Mann–Whitney test). MDA levels have been shown previously to be high in the plasma and serum from AD patients compared to age-matched control group [30], while an independent study also reported about unchanged plasma MDA level in AD [31]. In PD, significantly higher plasma MDA levels were previously found in the early-stages of the disease (Hoehn–Yahr stages I and II) compared to patients in later stages (stages III and IV) [32]. However, Sanyal et al. found no correlation between plasma MDA and age and discussed about unclear findings in the literature on these markers in PD [33]. In other words, it is unclear in the literature whether it is the analytical procedure or the type of assay that is responsible for such differences in the assessment of MDA. In our study, the plasma MDA was determined by using slightly modified protocol described previously, we therefore propose to re-evaluate the methodologies related to MDA-related biomarkers.

**Fig. 2:**
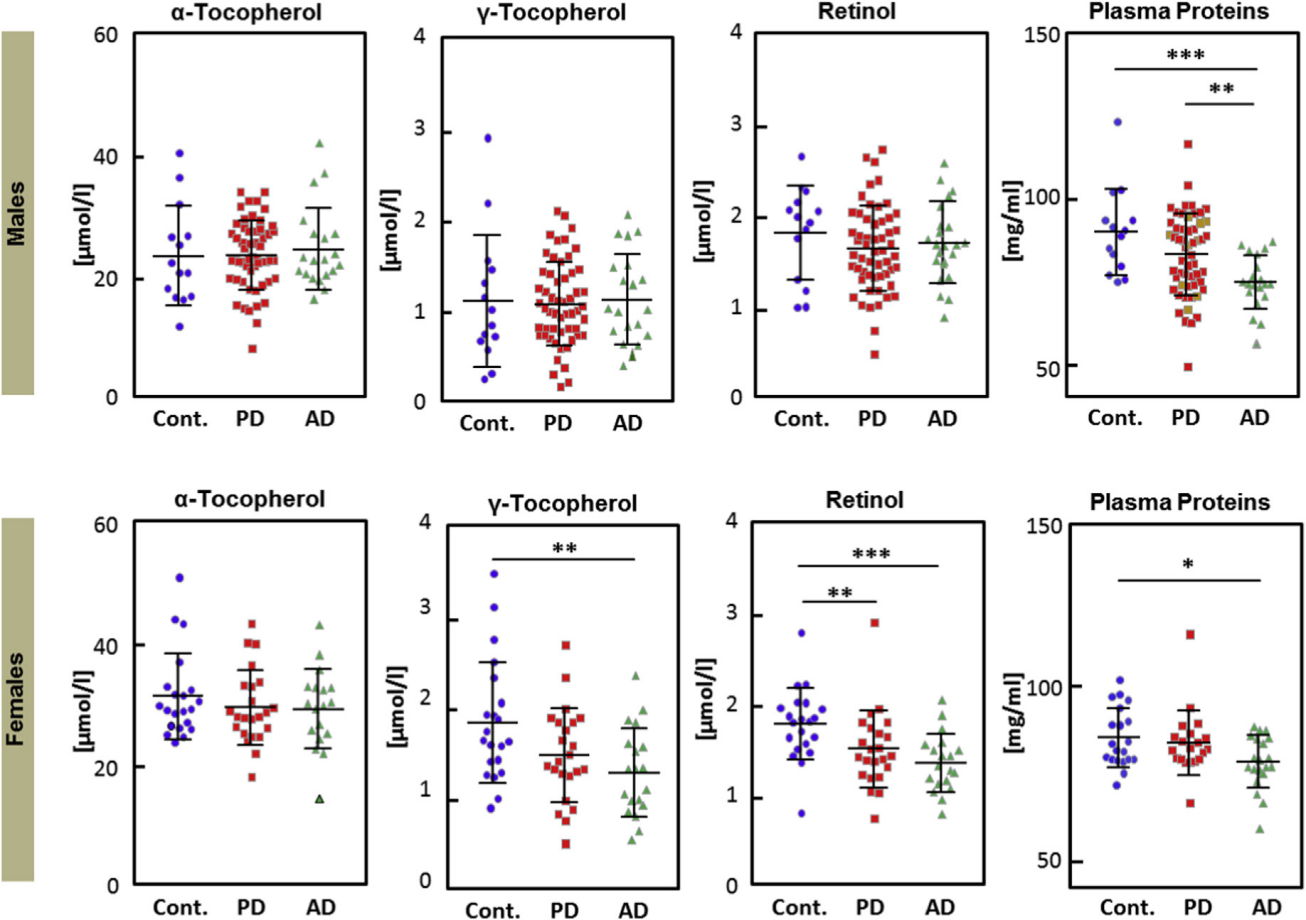
**Fat-soluble vitamins and plasma proteins**. Plots showing plasma levels α-tocopherol, γ-tocopherols, retinol and plasma proteins. Each dot represents one patient/participant. Asterisks indicate level of statistical significance (*p < 0.05, **p < 0.01, ***p < 0.001, ****p < 0.0001). [Cont: controls; AD: Alzheimer's disease, PD: Parkinson's disease].

For 3NT (3-Nitrotyrosine), we found differences only in males compared to HCs (Cont vs AD: p = 0,0134; PD vs AD p = 0,0256, Mann*–*Whitney test), male AD patients showed higher values but no such differences were observed in females, as confirmed by an independent previous study [34]. Given that diabetes is one of the major contributing factors for AGEs, we have limited information about PD samples and no data were available for the AD group. Importantly, we have not observed any inter-individual variations in the PD male samples with/without diabetes (marked in Fig. 1, Fig. 2). Also, the prevalence of diabetes is around 10% in the German population [35], and a similar proportion of patients with diabetes are present in our PD population.

### 3.2 Fat-soluble vitamins and carotenoids

Vitamin E (tocopherols) is considered an important antioxidant in the brain. While we did not observe significant differences in plasma levels of α–tocopherol, AD females did show significant differences in γ–tocopherol levels as compared to controls (p = 0,0068, Mann*–*Whitney test) (Fig. 2). Likewise, the retinol (Vitamin A) level was also found to be significantly lower in AD and PD females compared to healthy controls (p = 0,0xxx, Mann*–*Whitney test).

In case of plasma proteins, AD males and AD females showed significantly lower levels compared to the healthy control (Cont vs AD males: p = 0,0001; Cont vs AD females p = 0,0195, Mann*–*Whitney test). Also, the significant difference was observed between male AD and male PD samples for this particular marker (p = 0,0024, Mann*–*Whitney test). It is certainly worthwhile to investigate this question in a larger sample, as it might turn out that a lower total plasma protein could serve as a biomarker for AD. Also the redox state of plasma albumin (albumin turnover) which is primarily influenced by the dietary proteins [36] may play a role, but albumin synthesis rate was not measured in this study.

**Fig. 3:**
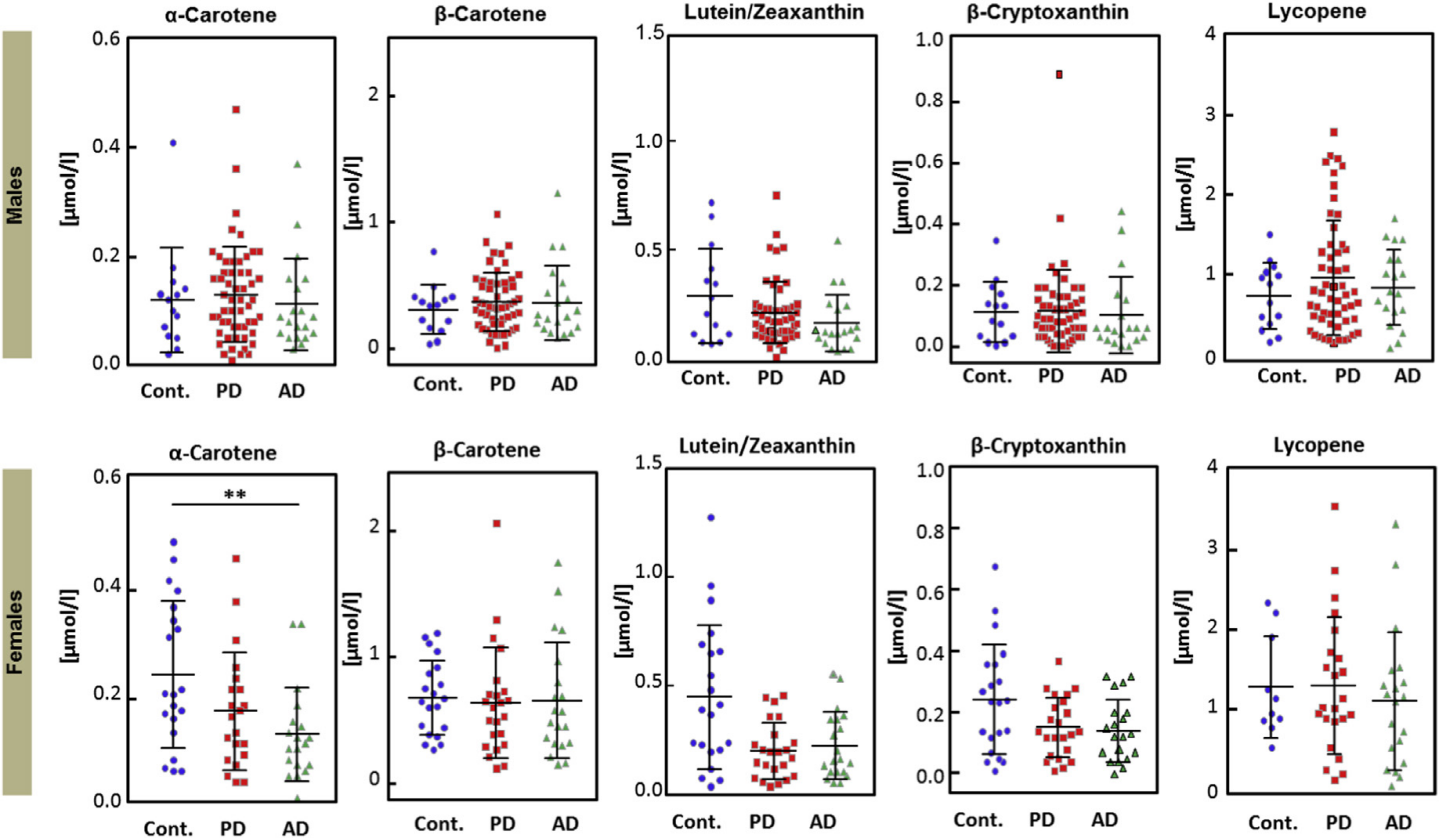
**Plasma carotenoids.** Plots showing plasma levels α-carotene, β-carotene, lutein/zeaxanthin, lycopene and β-cryptoxanthin. Each dot represents one patient/participant. Asterisks indicate level of statistical significance (*p < 0.05, **p < 0.01, ***p < 0.001, ****p < 0.0001). [Cont: controls; AD: Alzheimer's disease, PD: Parkinson's disease].

Among dietary micronutrients, α-carotene was lower in AD female patients compared to the controls (p = 0.0056, Mann–Whitney test). While β-carotene, lutein /zeaxanthin, β-cryptoxanthin and lycopene showed no significant differences between control and patients (Fig. 3). Interestingly, we observed higher concentrations of α-tocopherol (trend), γ-tocopherol, α- and β-carotene and β-cryptoxanthin in the control group in women (Table 1), which is in accordance with the MARK-AGE study, which was aimed to identify biomarkers of biological age and healthy aging [37]. It is also noteworthy to mention that given the rather small sample size investigated here we are critical with regard to the formal statistical significance but see a trend towards a reduced level of total plasma proteins in the AD population (Supplementary Table 1), which could well be related to either malnutrition due to dementia (secondary to the disease) or another metabolic phenomenon like Sarcopenia.

Oxidative stress is well accepted as an important pathogenic factor in AD and PD, largely based on the indirect evidences. Importantly, the differences between AD/PD and/or the sex specificity associated with increased oxidative stress in neurodegenerative diseases are not well understood. A possible factor could be the extensive use of animal models and/or lack of gender specific data from the body fluids. At the molecular level, the quantification of these oxidative stress related markers in the circulating/peripheral blood does not necessarily relate directly to their (presumed) functional aspect. However, it does point to the casual step towards the protein dysfunction and the subsequent consequences. Our data underline the need to carefully recognize sex-specific differences in all clinical investigations related to the identification of biomarkers for NDD.

## 4 Conclusion

Plasma levels of AGEs, protein carbonyls and several other biomarkers of oxidative stress (malondialdehyde, 3-nitrotyrosine, γ -tocopherols, retinol, plasma proteins and α -carotene) revealed sex-specific differences in AD and PD. Thus, we suggest that a clear distinction must be made in biomarker studies according to the sex. We also highlight the enrichment of protein-carbonyl motives in NDD associated proteins, which requires further attention. In future prospective, we discussed about the trend towards a reduced level of total plasma proteins mainly in the AD population, which could well be related to either malnutrition due to dementia (secondary to the disease) or another metabolic phenomenon like Sarcopenia.

## Declaration of competing interest

The authors declare no conflict of interests.

## Appendix A Supplementary data

Multimedia component 2 Multimedia component 2

Supplementary Fig. 1 **Carbonylation residues over NDD associated proteins.** The putative carbonylation residues over NDD associated proteins (*Tau, APP, α -Syn*) are labelled.*Tau*-*441*, the longest *Tau* isoform is also shown. Supplementary Fig. 1
