# Supplementary material for: Advanced glycation end products and protein carbonyl levels in plasma reveal sex-specific differences in Parkinson's and Alzheimer's disease
Source: Redox Biol. 2020 May 18;34:101546. doi: 10.1016/j.redox.2020.101546 (PMC7251371; doi:10.1016/j.redox.2020.101546)
Supplement: Multimedia component 1 [file mmc1.pdf]

Supplementary table 1. Sex and disease based variations in biomarker concentrations.

	Control		Parkinson´s		Alzheimer´s	
	Males (n=14)	Females (n=21)	Males (n=55)	Females (n=19)	Males (n=20)	Females (n=20)
**Age**	70.8 ± 12.1	66.6 ± 9.1	71.6 ± 8.8	70.2 ± 8.7	73.7 ± 4.2	72.0 ± 5.7
**CML (nmol/mg)**	52.4 ± 11.3	54.2 ± 14.7	74.0 ± 17.4	73.2 ± 17.5	79.2 ± 17.4	68.7 ± 14.9
**CEL (nmol/mg)**	23.70 ± 6.79	27.93 ± 5.13	23.27 ± 5.27	23.92 ± 6.89	22.59 ± 4.04	14.94 ± 3.83 §
**Protein Carbonyls (nmol/mg)**	0.04 ± 0.01	0.08 ± 0.04 §	0.05 ± 0.02	0.10 ± 0.07 §	0.07 ± 0.02	0.06 ± 0.02
**MDA (µmol/L)**	1.52 ± 0.81	1.74 ± 0.54	1.11 ± 0.63	1.18 ± 0.69	0.97 ± 0.23	1.01 ± 0.37
**3NT (pmol/mg)**	3.07 ± 2.94	4.69 ± 2.50	3.01 ± 1.27	2.65 ± 1.67	3.91 ± 1.45	3.49 ± 1.59
**α-Tocopherol (µmol/L)**	24.0 ± 8.1	32.0 ± 7.0 ¥	24.1 ± 5.6	30.2 ± 6.5	25.0 ± 6.7	34.3 ± 14.8
**γ-Tocopherol (µmol/L)**	1.13 ± 0.72	1.85 ± 0.66 §	1.10 ± 0.45	1.44 ± 0.52	1.15 ± 0.49	1.49 ± 0.94
**Retinol (µmol/L)**	1.84 ± 0.51	1.81 ± 0.39	1.66 ± 0.46	1.51 ± 0.45 ¥	1.73 ± 0.45	1.56 ± 0.85
**Plasma Proteins (mg/ml)**	89.3 ± 13.12	84.3 ± 8.9	82.6 ± 12.4	82.8 ± 9.9	74.0 ± 8.3	77.2 ± 8.1
**α-Carotene (µmol/L)**	0.12 ± 0.10	0.30 ± 0.32 §	0.13 ± 0.09	0.24 ± 0.23	0.11 ± 0.08	0.16 ± 0.17
**β-Carotene (µmol/L)**	0.30 ± 0.19	0.73 ± 0.45 §	0.37 ± 0.23	0.64 ± 0.47 ¥	0.36 ± 0.30	0.63 ± 0.46
**Lutein/Zeaxanthin (µmol/L)**	0.29 ± 0.22	0.45 ± 0.33	0.21 ± 0.14	0.23 ± 0.13	0.16 ± 0.13	0.23 ± 0.16
**β-Cryptoxanthin (µmol/L)**	0.11 ± 0.10	0.25 ± 0.18 §	0.11 ± 0.14	0.17 ± 0.09	0.10 ± 0.12	0.14 ± 0.10
**Lycopene (µmol/L)**	0.72 ± 0.41	1.10 ± 0.70	0.95 ± 0.72	1.29 ± 0.90	0.83 ± 0.47	1.07 ± 0.86

Table shows mean ± SD. § significant difference males vs. females ($p < 0.05$), ¥ trend ($p \leq 0.072$).). Only statistical differences between males and females of each group are shown in the table. Differences between sexes are shown in Figures 1-3.
