# Supplementary figures and images for: Advanced glycation end products and protein carbonyl levels in plasma reveal sex-specific differences in Parkinson's and Alzheimer's disease
Source: Redox Biol. 2020 May 18;34:101546. doi: 10.1016/j.redox.2020.101546 (PMC7251371; doi:10.1016/j.redox.2020.101546)

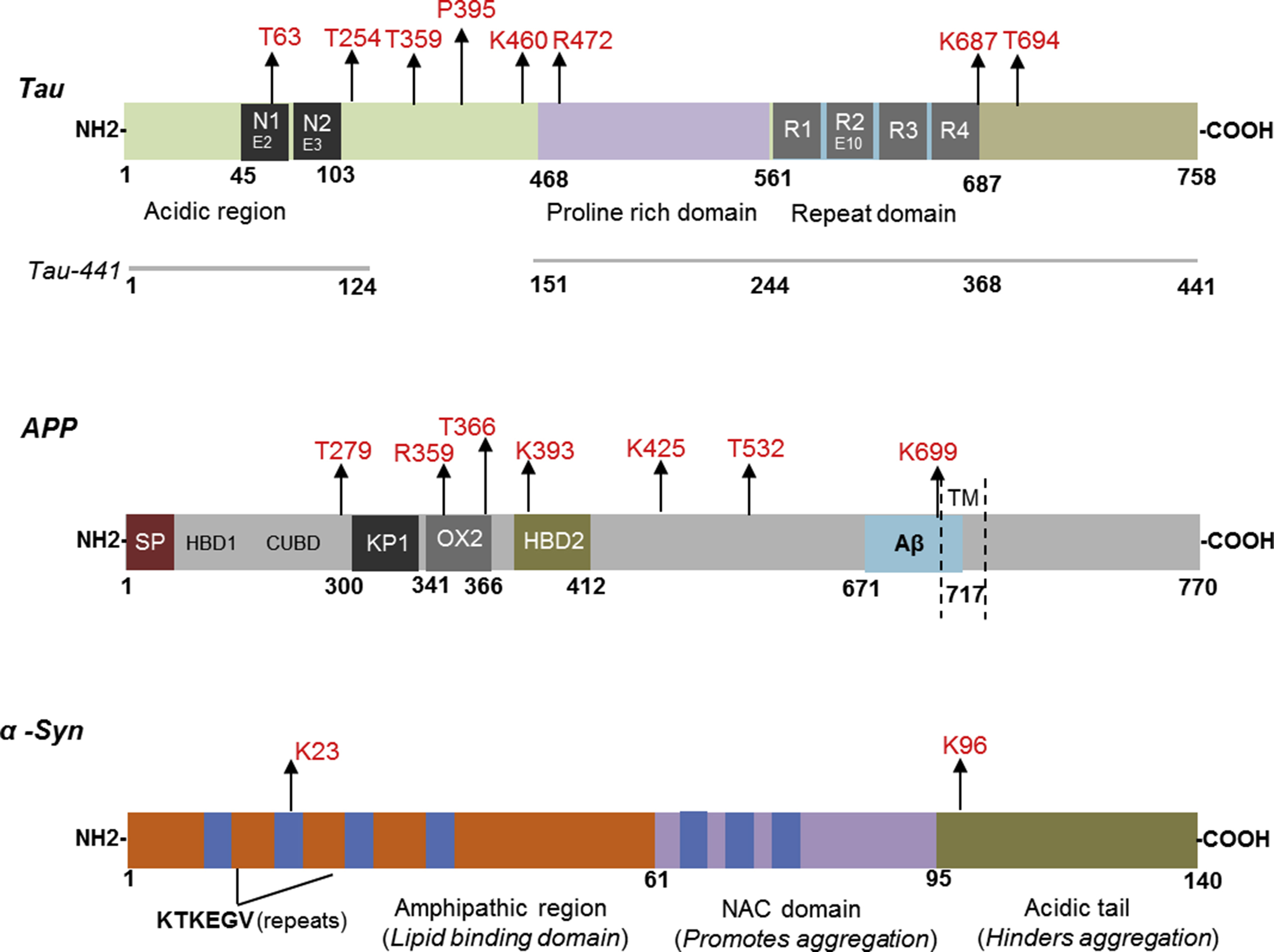

Supplement: Supplementary Fig. 1 — Carbonylation residues over NDD associated proteins. The putative carbonylation residues over NDD associated proteins (Tau, APP, α -Syn) are labelled.Tau-441, the longest Tau isoform is also shown. [file mmcfigs1.jpg]
